# New approaches to epidemic modeling on networks

**DOI:** 10.1038/s41598-022-19827-9

**Published:** 2023-01-10

**Authors:** Arturo Gómez, Gonçalo Oliveira

**Affiliations:** 1 Universidade Federal Fluminense, Rua Miguel de Frias, 9, Icaraí, Niterói, RJ 24220-900 Brasil; 2 IST Austria, Am Campus 1, 3400 Klosterneuburg, Austria; 3 Instituto Superior Tecnico, Lisboa, Portugal

**Keywords:** Network topology, Probabilistic data networks, Applied mathematics, Pure mathematics

## Abstract

In this article, we develop two independent and new approaches to model epidemic spread in a network. Contrary to the most studied models, those developed here allow for contacts with different probabilities of transmitting the disease (transmissibilities). We then examine each of these models using some mean field type approximations. The first model looks at the late-stage effects of an epidemic outbreak and allows for the computation of the probability that a given vertex was infected. This computation is based on a mean field approximation and only depends on the number of contacts and their transmissibilities. This approach shares many similarities with percolation models in networks. The second model we develop is a dynamic model which we analyze using a mean field approximation which highly reduces the dimensionality of the system. In particular, the original system which individually analyses each vertex of the network is reduced to one with as many equations as different transmissibilities. Perhaps the greatest contribution of this article is the observation that, in both these models, the existence and size of an epidemic outbreak are linked to the properties of a matrix which we call the ${\mathcal {R}}$-matrix. This is a generalization of the basic reproduction number which more precisely characterizes the main routes of infection.

## Introduction

### Context

A very natural way to model the spread of a human-to-human transmissible infectious disease is to encode each individual as the vertex of a graph whose edges model the interactions through which the disease can propagate. See^1–9^ and references therein for the vast literature of epidemic modeling, including on networks. We also refer the reader to some very interesting related work in^10–14^.

However, despite the large body of work, there are substantial difficulties in implementing such methods, the most obvious of which being the difficulty in inferring a realistic network and in analyzing the very high dimensional resulting system of ordinary differential equations. Furthermore, due to such difficulties most models make the additional simplifying assumption that all interactions have the same probability of transmitting the disease.

In fact, extending the theory in order to incorporate interactions with different probability of transmitting the disease, dealing with heterogeneity, developing approximation schemes, and understanding network based interventions are all listed as some of the main challenges facing network epidemic modeling as stated in^7^.

These challenges are in fact all linked as, for instance, understanding which interactions are the most responsible for the epidemic spread would allow for better insight on which kind of interventions are the most effective in controlling an outbreak. This is therefore a fundamental research direction which ought to be pursued with more intensity in the future.

In this work we shall use a method that at the same time deals with the two difficulties mentioned above while at the same time incorporating the possibility of different types of interactions. Our results are insightful and our techniques tractable enough so that they can be effectively used in the future in a large amount of situations.

### Summary of results

We shall now summarize our approach and main results. Consider a large number of individuals $N\in {\mathbb {N}}$ interacting with each other through $n \in {\mathbb {N}}$ different types of interactions which have probabilities $T_1, \ldots ,T_n \in [0,1]$ of transmitting the disease (typically $1 \le n \ll N$). To encode the network we use *n* different graphs ${\mathcal {G}}_1, \ldots , {\mathcal {G}}_n$ whose edges represent the different interactions and ${\mathcal {G}}={\mathcal {G}}_1 \cup \cdots \cup {\mathcal {G}}_n$ is the total graph. For example, an edge of the graph ${\mathcal {G}}_i$ encodes an interaction which has a probability $T_i$ of transmitting the disease.

In reality, we shall require relatively little information on the specific properties of the network encoding the interactions. Namely, we will only need to know the degree distributions for the several types of interactions. Said in other words, we require knowledge of the probability that a randomly chosen vertex has a certain number of interactions of each type (notice that this is far less than knowing the exact form of the network).

#### Summary of methodology and main contribution

The key technical method which we employ to deal with this is to use multivariate generating functions in order to simplify computations and have a unified approach which only depend on the degree distributions of the graphs encoding the network. Therefore, we start in section “Generating functions” by recalling the definition multivariate generating functions for the excess degree distributions following a random edge of ${\mathcal {G}}_i$, for $i=1, \ldots ,n$. These are denoted by ${\tilde{G}}_i(x_1,\ldots ,x_n)$ and we further use them to construct a $n \times n$ matrix with entries

$$\begin{aligned} {\mathcal {R}}_{ij}^{{\tilde{G}}}= T_j \frac{\partial {\tilde{G}}_i}{\partial x_j} \Bigg \vert _{x_1=\cdots =x_n=1}, \end{aligned}$$

which we shall call the ${\mathcal {R}}^{{\tilde{G}}}$-matrix. This matrix has several interesting properties and, as will become later clear, encodes much epidemic information. For example, the sum of all entries in the *i*-th line $R_0(i)=\sum _{j}{\mathcal {R}}_{ij}^{{\tilde{G}}}$ coincides with the basic reproduction number of infections caused by the individuals that originally got infected through an interaction of ${\mathcal {G}}_i$. Also, the total basic reproduction number can be easily recovered from ${\mathcal {R}}_{ij}^{{\tilde{G}}}$ as shown in Remark 8.

Still in section “Generating functions” we define some modified generating functions, which we call $\tilde{H_i}(x_1, \ldots , x_n)$, and use to construct a ${\mathcal {R}}^{{\tilde{H}}}$-matrix similarly.

Section “Prevalence of infection and percolation (the late stages of an outbreak)” constructs the first, and most basic, of our approximate models. This looks at the late stages of an epidemic outbreak that propagated in ${\mathcal {G}}$ and assumes that the disease already had enough time to sufficiently spread through in the population and came to some sort of equilibrium with part of the population being removed after infection and transmission. We employ this into a mean field type approximation which, in particular, implies that all individuals with the same number of interactions of each kind, have the same probability of already having been infected. Our analysis can also be considered as a model of percolation with *n* different types of nodes. This important problem in itself, seems to not have received much attention, see^15^ for a honorable exception.

Our findings, stated in Propositions 1, 2, and 3, relate the existence of a phase transition in the fraction of infected vertices and the eigenvalues of the ${\mathcal {R}}^{{\tilde{G}}}$-matrix crossing the value 1.

Section “Dynamic modeling in a local mean field approximation” lays out an enormous system of ordinary differential equations (ODEs) modeling a SIR-type epidemic dynamically spreading in a network encoded by ${\mathcal {G}}$. This model generalizes the more standard version by allowing for different transmissibilities depending on which graph ${\mathcal {G}}_i$ encodes a specific interaction. In total, this results in a system of *N* ODEs for *N* unknown functions.

The analysis of this model is mostly postponed until section “Dynamic modeling in a mean field approximation by degree similarity” , with 4 simply proving that as $t \rightarrow +\infty$, the system converges to a disease free equilibrium for which some characterizations are given. For example, Theorem 1 shows that a vertex $v \in {\mathcal {G}}$ has a probability of at most $1-\exp (-R(v))$ of ever being infected, where *R*(*v*) denotes the number of infections *v* is expected to cause if infected. Interestingly, this simple bound highlights that individuals expected to infect many others are also more likely to be infected.

Section “Dynamic modeling in a mean field approximation by degree similarity” further analyses the previous system of ODEs by making one extra simplifying assumption. Namely, that vertices with the same joint degree have similar probabilities of being in each state. We then show that such a simplifying assumption reduces the original system of *N* ODEs to a much smaller one of *n* ODEs. In this situation, we find that the matrix ${\mathcal {R}}^{{\tilde{H}}}$ is of fundamental importance in understanding the dynamics of the epidemic outbreak. For example, we prove that the existence of an eigenvalue greater than one is related with the nearly exponential growth of the outbreak in its early stages.

#### A comment on computational methods

Before embracing in proving the main results we want to make one further comment. There are computational tools which can be used to implement epidemics spreading on networks such as the EoN and SEIRSplus Python packages. While these do exemplify the well known epidemic behaviors that we describe, our main contribution is to rigorously mathematically demonstrate the mentioned results using the very general models we consider (recall that we allow for $n \ge 1$ different transmissibilities). This goes beyond the current state of the art as these results had only been rigorously established in the case $n=1$.

## Generating functions

This section reviews some basics of generating functions, including multivariate generating functions. For some fascinating early and varied applications of the method of generating functions we recommend^16^.

### Graphs and their generating functions

Let ${\mathcal {G}}$ be a graph whose vertices $v({\mathcal {G}})$ encode individuals and whose edges $e({\mathcal {G}})$ encode interactions through which the disease can spread. We consider a family of $n \in {\mathbb {N}}$ subgraphs ${\mathcal {G}}_1 , \ldots , {\mathcal {G}}_n \subset {\mathcal {G}}$ with $v({\mathcal {G}}_i)=v({\mathcal {G}})$, $e({\mathcal {G}}_i) \subset e({\mathcal {G}})$ for $i=1,\ldots ,n$ and $e({\mathcal {G}}_1) \cup \cdots \cup e({\mathcal {G}}_n) = e({\mathcal {G}})$. For each $i=1, \ldots , n$, let $\lbrace P_i(k) \rbrace _{k \in {\mathbb {N}}_0}$ be the degree distribution of the graph ${\mathcal {G}}_i$ meaning that a randomly chosen vertex has degree *k* with probability $P_i(k)$. The corresponding generating functions are given by

$$\begin{aligned} G_i(x) = \sum _{k=0}^{+\infty } P_i(k) x^k, \quad \text {for } i=1, \ldots , n, \end{aligned}$$

and we define the joint multivariate generating function by

$$\begin{aligned} G(x_1 , \ldots , x_n) = \sum _{k_1=0}^{+\infty } \ldots \sum _{k_n=0}^{+\infty } \prod _{i=1}^{n} P_i (k_i) x_i^{k_i} = \prod _{i=1}^{n} G_i (x_i). \end{aligned}$$

#### Remark 1

Notice that $G_i'(x)=\sum _{k=1}^{+\infty } kP_i(k) x^{k-1}$ and so the average degree of ${\mathcal {G}}_i$, denoted $\mathbf{E}(k_i)$, may be computed to be

$$\begin{aligned} {\mathbf{E}}(k_i)= G_i'(1). \end{aligned}$$

Similarly, using the fact that $G_i(1)=1$, we find the total average degree to be

$$\begin{aligned} {\mathbf{E}}(k)= \sum _{i=1}^n G_i'(1) = \frac{d}{dx} G(x , \ldots , x) \Big \vert _{x=1}. \end{aligned}$$

#### Remark 2

The idea of having the *n* subgraphs ${\mathcal {G}}_1 , \ldots , {\mathcal {G}}_n$ is that of modeling different types of contacts between individuals which have unequal probability of transmitting the disease. Thus, we associate a probability of transmission $T_i$ to each $i=1, \ldots , n$ and assume with no loss of generality that $T_1> \cdots > T_n$.

### Excess degree distributions

Consider a randomly chosen individual which may have been infected following a randomly chosen transmission. Considering it as a vertex in ${\mathcal {G}}_i$, we define its excess degree (or ramification) as the number of extra edges emanating from it. The joint probability that such a vertex has ramification $(k_1 , \ldots , k_n)$ along the graphs ${\mathcal {G}}_1, \ldots , {\mathcal {G}}_n$ can be computed directly from the degree distributions $P_i(\cdot )$ of the graphs ${\mathcal {G}}_i$ as in^17^. Indeed, given that fixing the subgraph ${\mathcal {G}}_i$ there are $k_i+1$ ways of arriving at a vertex with degree $k_i+1$ (ramification $k_i$) we find that

$$\begin{aligned} {\tilde{P}}(k_1 , \ldots , k_n) = \frac{ \sum _{l=1}^n(k_l+1)P_l(k_l+1) \prod _{i \ne l}P_i(k_i) }{\mathbf{E}(k)}. \end{aligned}$$

This will be referred to as the excess degree, or ramification, joint distribution.

#### Remark 3

Here we are working with the excess degree distribution for a randomly chosen individual rather than than the excess degree distribution for a randomly chosen infected individual.

The associated multivariate generating function

1 $$\begin{aligned} {\tilde{G}}(x_1, \ldots , x_n) : = \sum _{k_1 , \ldots , k_n} {\tilde{P}}(k_1 , \ldots , k_n) x_1^{k_1} \ldots x_n^{k_n}, \end{aligned}$$

which as shown in^17^ can be written in terms of the $G_i(x_i)$ for $i=1, \ldots , n$ as follows

2 $$\begin{aligned} {\tilde{G}}(x_1, \ldots , x_n) = \frac{\sum _{l=1}^n G_l'(x_l) \prod _{i \ne l}G_i(x_i)}{\sum _{l=1}^n G_l'(1) } , \end{aligned}$$

and recall that ${\mathbf{E}}(k)=\sum _{l=1}^n G_l'(1)$.

#### Remark 4

Let $m_1, \ldots , m_n \in {\mathbb {N}}_0$ with $m_1 + \cdots + m_n >0$ and consider a randomly chosen individual *v* and assume that the remaining population is all susceptible. Using the distribution for the excess ramification, the probability that, if infected, *v* will infect $m_i$ individuals along ${\mathcal {G}}_i$ for each *i*

3 $$\begin{aligned} p(m_1 , \ldots , m_n) = \sum _{k_1 \ge m_1 , \ldots , k_n \ge m_n} {\tilde{P}}(k_1 , \ldots , k_n) \prod _{i=1}^n {k_i \atopwithdelims ()m_i} T_i^{m_i} (1-T_i)^{k_i-m_i} . \end{aligned}$$

Then, the generating function for the random variable *M* given by the number of infections caused by a randomly chosen individual, if infected, is

4 $$\begin{aligned} \begin{aligned} G_M(x)&= \sum _{m_1, \ldots , m_n} p(m_1 , \ldots , m_n) x^{m_1 + \cdots + m_n} \\&= \sum _{k_1 , \ldots , k_n} \sum _{m_1=0, \ldots , m_n=0}^{k_1 , \ldots , k_n} {\tilde{P}}(k_1 , \ldots , k_n) \prod _{i=1}^n {k_i \atopwithdelims ()m_i} (xT_i)^{m_i} (1-T_i)^{k_i-m_i} \\&= \sum _{k_1, \ldots , k_n} {\tilde{P}}(k_1 , \ldots , k_n) \prod _{i=1}^n (xT_i+1-T_i)^{k_i} \\&= {\tilde{G}}(1+T_1(x-1) , \ldots , 1+ T_n(x-1)). \end{aligned} \end{aligned}$$

#### Remark 5

Recall that, for a disease starting to propagate in an otherwise completely susceptible population, the basic reproduction number$R_0$ is given by $R_0=G_M'(1)$, which from the above formula can be computed to be

$$\begin{aligned} R_0&= \sum _{j=1}^n T_j \frac{\partial {\tilde{G}}}{\partial x_j} \Big \vert _{x_1= \cdots = x_n=1}. \end{aligned}$$

### Specific excess degree distributions

We now consider the excess degree distribution by following an edge of a specific graph ${\mathcal {G}}_i$, we define its available ramification as the number of excess edges emanating from it. Then, as before, the joint probability that such a vertex has excess degree $(k_1 , \ldots , k_n)$ is

$$\begin{aligned} {\tilde{P}}_i(k_1 , \ldots , k_n) = \frac{(k_i+1)P_i(k_i+1) \prod _{j \ne i}P_j(k_j) }{{\mathbf{E}}(k_i)}. \end{aligned}$$

Therefore, its multivariate generating function ${\tilde{G}}_i(x_1,\ldots , x_n)= \sum _{k_1, \ldots , k_n} {\tilde{P}}_i(k_1, \ldots ,k_n) x_1^{k_1} \ldots x_n^{k_n}$ can be computed to be

5 $$\begin{aligned} {\tilde{G}}_i(x_1,\ldots , x_n)= \frac{G_i'(x_i)}{G_i'(1)} \prod _{j \ne i}G_j(x_j) . \end{aligned}$$

#### Remark 6

Notice, in particular that ${\tilde{P}}(k_1 , \ldots , k_n)= \sum _{i=1}^n \frac{{\mathbf{E}}(k_i)}{{\mathbf{E}}(k)} {\tilde{P}}_i(k_1 , \ldots , k_n)$. Using either this fact or the previous formulas for the generating functions we find that ${\tilde{G}}$ can be computed from the $\tilde{G_i}$ as follows

$$\begin{aligned} {\tilde{G}}(x_1,\ldots , x_n)= \frac{\sum _{l=1}^n G_l'(1) {\tilde{G}}_l(x_1 , \ldots , x_n)}{\sum _{l=1}^n G_l'(1)}. \end{aligned}$$

#### Definition 1

Let $i=1, \ldots ,n$ and $\tilde{G_i}(x_1, \ldots , x_x)$ be as in (5). Define the ${\mathcal {R}}^{{\tilde{G}}}$-matrix as the $n \times n$ matrix whose (*i*, *j*) entries are

$$\begin{aligned} {\mathcal {R}}^{{\tilde{G}}}_{ij}:= T_j\frac{\partial {\tilde{G}}_i}{\partial x_j} \Big \vert _{x_1=\cdots = x_n=1}. \end{aligned}$$

Furthermore, given $s_{(k_1, \ldots k_n)}\in [0,1]$ for all $(k_1, \ldots k_n) \in {\mathbb {N}}_0^n$ and $i=1, \ldots , n$ we shall define the generating function

6 $$\begin{aligned} \tilde{H_i}(x_1, \ldots , x_n) = \sum _{k_1, \ldots , k_n} {\tilde{P}}_i(k_1, \ldots ,k_n) s_{(k_1, \ldots , k_i+1 , \ldots k_n)} x_1^{k_1} \ldots x_i^{k_i+1} \ldots x_n^{k_n}. \end{aligned}$$

It will prove useful to also define a ${\mathcal {R}}$-matrix associated with these generating functions as follows.

#### Definition 2

Let $s= \lbrace s_{(k_1, \ldots k_n)}\in [0,1] \ | \ \ (k_1, \ldots k_n) \in {\mathbb {N}}_0^n \rbrace$ and for $i=1, \ldots ,n$ the function $\tilde{H_i}(x_1, \ldots , x_x)$ be as in (6). Then, we define the $n \times n$ matrix whose (*i*, *j*) entries are

$$\begin{aligned} {\mathcal {R}}^{{\tilde{H}}}_{ij}:= T_j\frac{\partial {\tilde{H}}_i}{\partial x_j} \Big \vert _{x_1=\cdots = x_n=1}, \end{aligned}$$

which we shall call the ${\mathcal {R}}^{{\tilde{H}}}$-matrix.

#### Remark 7

In the case when $s_{(k_1, \ldots , k_n)}=1$ we have $\tilde{H_i}=x_i\tilde{G_i}$ and so the ${\mathcal {R}}^{{\tilde{H}}}$-matrix turns into

$$\begin{aligned} {\mathcal {R}}^{{\tilde{H}}}_{ij}:= T_i \delta _{ij} + T_j\frac{\partial {\tilde{G}}_i}{\partial x_j} \Big \vert _{x_1=\cdots = x_n=1} = T_i \delta _{ij} + {\mathcal {R}}^{{\tilde{G}}}_{ij} . \end{aligned}$$

#### Remark 8

We can define a quantity $R_0(i)$ which yields the average number of infections caused by the individuals which were themselves infected from an interaction of ${\mathcal {G}}_i$. In formulas, such a quantity is given by

7 $$\begin{aligned} R_0(i):= \sum _{j=1}^n T_j \frac{\partial \tilde{G_i}}{\partial x_j} \Big \vert _{x_1= \cdots = x_n=1}, \end{aligned}$$

or, in terms of the first reproduction matrix, as the sum of all the entries in the i-th line, i.e. $R_0(i)= \sum _{j=1}^n {\mathcal {R}}^{{\tilde{G}}}_{ij}$. It is easy to check that

$$\begin{aligned} R_0 = \sum _{i=1}^n \frac{{\mathbf{E}}(k_i)}{{\mathbf{E}}(k)} R_0(i). \end{aligned}$$

## Prevalence of infection and percolation (the late stages of an outbreak)

Recall that for $i=1, \ldots , n$ the transmissibilities $T_i$ denote the probability that an interaction, encoded by an edge of ${\mathcal {G}}_i$, will transmit the disease if one of its ends is infected. We point out that this approach is in different from that of multiplex networks as in that case it is the different nodes that have different transmissibilities, see for example^18–20^. In our case each node can have several $T_i$ and so our approach can be interpreted as a generalization of the multiplex networks case. We also point out that we shall work in full generality and our results are widely applicable.

At this point we follow a well known trick first introduced in^1^ and^21^ in the case when there is only one transmissibility. In order to implement this trick in the case when there is more than one transmissibility we introduce *n* quantities, called $q_1, \ldots , q_n$: Each $q_i$ corresponds to the average probability that a vertex is not infected through a specific interaction (edge) of ${\mathcal {G}}_i$. For this to happen, either:

- the infection is not transmitted (independently of whether the individual in the other end of this interaction is infected or not) which has probability $1-T_i$, or
- the infection would be transmitted by the interaction, with probability $T_i$, but the other individual was not infected, which happens with probability $\prod _{j=1}^n q_j^{k_j}$ if it has excess degree $(k_1, \ldots , k_n)$.

Hence, on average we have

8 $$\begin{aligned} q_i = 1- T_i + T_i \sum _{k_1 , \ldots , k_n} {\tilde{P}}_i(k_1 , \ldots , k_n) \prod _{j=1}^n q_j^{k_j} = 1-T_i + T_i {\tilde{G}}_i(q_1, \ldots , q_n), \end{aligned}$$

for $i=1, \ldots , n$. This is a fixed point equation for the function $F(q_1, \ldots , q_n)=(F_1 , \ldots , F_n)$ with $F_i(q_1, \ldots , q_n)$ given by the right hand side of Eq. (8).

Then, the average probability that a randomly chosen vertex does not get infected is given by

9 $$\begin{aligned} {\mathbf{P}}= \sum _{k_1 , \ldots , k_n} P(k_1 , \ldots , k_n) q_1^{k_1} \ldots q_n^{k_n} = G(q_1, \ldots , q_n). \end{aligned}$$

### Remark 9

Notice that the probability a vertex with degree $(k_1, \ldots , k_n)$ does not get infected is given by

$$\begin{aligned} {\mathbf{P}}_{(k_1, \ldots , k_n)} = q_1^{k_1} \ldots q_n^{k_n} , \end{aligned}$$

and so ${\mathbf{P}}$ is simply the average of these probabilities. So, we see that this mean field type approximation also implies the weaker approximation where the probability that a vertex gets or not infected only depends on its degree.

We turn now to the question of finding conditions which guarantee that (8) has a solution other than the obvious one at $(1, \ldots , 1)$, which corresponds to the absence of disease. Before we embrace in the general analysis we consider the simple special case when $n=1$ which already appears in the literature, for example chapter 16 in^2^.

### Example 1

In the case when $n=1$ the fixed point equation (8) reads

10 $$\begin{aligned} q= 1-T + T {\tilde{G}}(q). \end{aligned}$$

Denoting the right hand side by *F*(*q*), we have $F(1)=1$ and $F(0)=1-T+T{\tilde{G}}(0)\ge 1-T>0$ while $F'(q)=T {\tilde{G}}'(q)>0$ and $F''(q)=T {\tilde{G}}''(q) \ge 0$. It then follows from the intermediate value theorem that there is a fixed point $q \in (0,1)$ of *F* if and only if $F'(1)>1$. Such a condition is given by $T {\tilde{G}}'(q)>1$ which can equally be written as $R_0>1$.

In this simple setting when $n=1$, we can further try to better understand the transition phenomena at $T=T_c$ such that $T_c {\tilde{G}}'(1)=1$. For this we expand ${\tilde{G}}$ around $q_c=1$ as a Taylor series

$$\begin{aligned} {\tilde{G}}(x)=1 + {\tilde{G}}'(1) (1-q) + \frac{{\tilde{G}}''(1)}{2} (1-q)^2 + \cdots , \end{aligned}$$

and inserting into Eq. (10) we have

$$\begin{aligned} q&= 1-T+ T \left( 1 + {\tilde{G}}'(1) (q-1) + \frac{{\tilde{G}}''(1)}{2} (q-1)^2\right) + \cdots \\&= 1 + (q-1) \frac{T}{T_c} + (q-1)^2 \frac{T{\tilde{G}}''(1)}{2} + \cdots , \end{aligned}$$

where we have used ${\tilde{G}}'(1)=T_c^{-1}$. This can be rewritten as

$$\begin{aligned} 1&= \frac{T}{T_c} - (1-q) \frac{T{\tilde{G}}''(1)}{2} , \end{aligned}$$

from which we find

$$\begin{aligned} q= 1- \frac{2}{T{\tilde{G}}''(1)} \left( \frac{T}{T_c} - 1 \right) + \cdots . \end{aligned}$$

Then, expanding ${\mathbf{P}}$ in a Taylor series around $q_c=1$ we find

11 $$\begin{aligned} {\mathbf{P}} &= 1 + (q-1) G'(1) + \cdots \nonumber \\&= 1 - \frac{2G'(1)}{{\tilde{G}}''(1)} \frac{1}{T} \left( \frac{T}{T_c} - 1 \right) + \cdots , \end{aligned}$$

valid for $T\ge T_c$ and which describes the phase transition as a power law with exponent 1.

Continuing to explore the case when $n=1$ we shall now give two very simple examples which can be solved explicitly.

### Example 2

(*2 neighbors and* $n=1$) In this situation each individual contacts with only two other ones, we have $G(x)=x^2$ and ${\tilde{G}}(x)=x$. Then, the fixed point equation is $q= 1-T + T q$ which has the unique solution $q=1$ independently of $T \in (0,1)$. The only other solution is $q=0$ which occurs in the case when $T=1$.

This is to be expected as if there is a probability that the interactions will not transmit the disease, then almost surely there will be someone which does not transmit it and so it does not get passed that individual. In a large population, almost everyone will be left uninfected.

### Example 3

(*3 neighbors and* $n=1$) In this example we consider $n=1$ and $G(x)=x^3$ so ${\tilde{G}}(x)=x^2$. Then, the fixed point equation turns into $q= 1-T + T q^2$. The only solution $T \in (0,1)$ is given by

$$\begin{aligned} q=\frac{1- |2T-1|}{2T} = \left\{ \begin{array}{ll} 1 , &{}\quad \text {if } T \le \frac{1}{2}, \\ \frac{1-T}{T} , &{}\quad \text {if } T > \frac{1}{2}. \end{array}\right. \end{aligned}$$

Hence, we see an interesting explicit phase transition occurring at $T=T_c=1/2$. In terms of the probability ${\mathbf{P}}$ that a randomly chosen individual escapes infection we find that

$$\begin{aligned} {\mathbf{P}}= \left\{ \begin{array}{ll} 1 , &{}\quad \text {if } T \le \frac{1}{2}, \\ \left( \frac{1-T}{T} \right) ^3 , &{}\quad \text {if } T > \frac{1}{2}. \end{array}\right. \end{aligned}$$

Notice that this is compatible with Eq. (11). Indeed, expanding ${\mathbf{P}}$ near $T_c=1/2$ we find that ${\mathbf{P}}= 1-12(T - T_c) + \cdots$.

When $n \ge 1$ we can equally prove existence of a critical point in $(0,1)^n$ if certain *n* quantities are greater than one (in the $n=1$ case there is a single quantity which can be readily identified with $R_0>1$). However, in this more general case the proof is slightly less elementary as this is a codimension $n>1$ problem for which the intermediate value theorem no longer applies.

On possible approach would be to denote by $F:[0,1]^n \rightarrow [0,1]^n$ the function defined by the right hand side of (8) and find hypothesis so that there is $\delta >0$ such that $F([0,1-\delta ]^n) \subset [0,1-\delta ]^n$. Then, the Brower fixed point theorem would guarantee the existence of such a fixed point in $(0,1)^n$. However, we shall instead proceed in a slightly different manner.

### Proposition 1

*Suppose that for all* $i=1, \ldots , n$, *the quantities* $R_0(i)$ *defined in* (7) *are all greater than* 1, *i.e.*

$$\begin{aligned} R_0(i) = \sum _{j=1}^n T_j \frac{\partial {\tilde{G}}_i}{\partial q_j} \Big \vert _{q_1= \cdots = q_n=1}>1. \end{aligned}$$

*Then, there is a solution* $(q_1 , \ldots , q_n) \in (0,1)^n$ *of Eq.* (8) *and so* ${\mathbf{P}}\in (0,1)$.

### Proof

We shall look for solutions of (8) in $(0,1)^n$ and it will prove convenient to write these as $q_i=1-T_i \varepsilon _i$. Then, the fixed point equation (8) turns into the equations

$$\begin{aligned} \varepsilon _i = 1- {\tilde{G}}_i(1-T_1 \varepsilon _1 , \ldots , 1-T_n \varepsilon _n), \quad {i=1, \ldots , n}, \end{aligned}$$

for $\varepsilon =(\varepsilon _1, \ldots , \varepsilon _n)$. Then, we look for fixed points of the function *F* given by the right hand side of equation above with $\varepsilon \ne 0$. By Brower’s fixed point theorem, for such a fixed point to exist, it is enough if $F: [0,T_1^{-1}] \times \cdots [0,T_n^{-1}] \rightarrow [0,1]^n$ maps $[\delta ,1]^n$ to itself, for some positive $\delta \ll 1$. First, notice that each entry

$$\begin{aligned} F_i(\varepsilon _1 , \ldots , \varepsilon _n) = 1- {\tilde{G}}_i(1-T_1 \varepsilon _1 , \ldots , 1-T_n \varepsilon _n), \end{aligned}$$

is nondecreasing in each coordinate and its image lies in $[0,1]^n$. Furthermore, by Taylor’s formula

$$\begin{aligned} F_i(\varepsilon _1, \ldots , \varepsilon _j ) = \sum _{j=1}^n T_j \frac{\partial {\tilde{G}}_i}{\partial x_j} \Big \vert _{x_1 = \cdots =x_n=1} \varepsilon _j + o(|\varepsilon |), \end{aligned}$$

for $|\varepsilon |\ll 1$. This shows that for sufficiently small $\delta >0$

$$\begin{aligned} F_i(\delta , \ldots , \delta ) = \left( \sum _{j=1}^n T_j \frac{\partial {\tilde{G}}_i}{\partial x_j} \Big \vert _{x_1 = \cdots =x_n=1} \right) \delta + o(\delta ) > \delta , \end{aligned}$$

if

$$\begin{aligned} \sum _{j=1}^n T_j \frac{\partial {\tilde{G}}_i}{\partial x_j} \Big \vert _{x_1 = \cdots =x_n=1} >1 . \end{aligned}$$

The quantities in the left hand side can be readily identified with the $R_0(i)$, from which we conclude that under these hypothesis $F([\delta ,1]^n) \subset [\delta ,1]^n$ and a fixed point exists. $\square$

Inspired by this proof and the computation in example 1, also for $n \ge 1$ we shall search for a phase transition (or bifurcation) from $q_c=(1, \ldots ,1)$ due to a variation in the parameters $T=(T_1, \ldots ,T_n)$. In order to set up the nomenclature, we shall consider a 1-parameter family $t \in I \subset {\mathbb {R}} \mapsto T(t)$ of transmissibilities. For all $t \in I$ we have that $q_c=(1, \ldots , 1)$ is a solution to (8). We shall say that a bifurcation from $q_c$ occurs at *T*(0) if any neighborhood of $(q_c, T(0))$ in ${\mathbb {R}}^n \times I$ contains solutions of (8) not equal to $q_c$. This will be called a phase transition if such solutions lie in a continuous curve parameterized by *t*. The following result gives a necessary condition for the existence of a phase transition.

### Proposition 2

*Consider a* 1-*parameter family of parameters* $T(t)=(T_1(t), \ldots , T_n(t))$ *and suppose that there is a continuous* $t \mapsto q(t)=(q_1(t), \ldots , q_n(t))$ *solution to* (8), *for* $t \in (-\delta , \delta )$ *with* $q(t)=(1, \ldots , 1)$ *for* $t \le 0$ *and* $q(t) \in (0,1)^n$ *for* $t>0$. *Then, the* ${\mathcal {R}}^{{\tilde{G}}}$-*matrix at* *T*(0) *must have* 1 *as one of its eigenvalues*.

### Proof

Bifurcations of *q* from $q_c$ at *T*(0) are in one to one correspondence with bifurcations of $\varepsilon$ from $\varepsilon _c=0$ at *T*(0). Defining the function ${\mathcal {G}}=({\mathcal {G}}_1, \ldots , {\mathcal {G}}_n)$ whose entries are

$$\begin{aligned} {\mathcal {G}}_i(\varepsilon _1, \ldots , \varepsilon _n)= 1- {\tilde{G}}_i(1-T_1 \varepsilon _1 , \ldots , 1-T_n \varepsilon _n) - \varepsilon _i, \end{aligned}$$

for $i=1, \ldots , n$. By the implicit function theorem, if for a given *T* we had $d{\mathcal {G}}_0: {\mathbb {R}}^n \rightarrow {\mathbb {R}}^n$ being an isomorphism, then no bifurcation could occur. This already gives us a necessary condition for a bifurcation to occur.

The (*i*, *j*) entry of $d{\mathcal {G}}_0$ is given by

12 $$\begin{aligned} \frac{\partial {\mathcal {G}}_i}{\partial \varepsilon _j}\Big \vert _{\varepsilon =0} = T_j \frac{\partial {\tilde{G}}_i}{\partial x_j} \Big \vert _{x=1} - \delta _{ij} = {\mathcal {R}}^{{\tilde{G}}}_{ij}-\delta _{ij}, \end{aligned}$$

and so $d{\mathcal {G}}_0$ is non-invertible if and only if the ${\mathcal {R}}^{{\tilde{G}}}$-matrix has 1 as an eigenvalue. We have thus concluded that for a bifurcation to occur at a given *T*, the ${\mathcal {R}}^{{\tilde{G}}}$-matrix must have a unit eigenvalue. $\square$

### Remark 10

We also mention in passing that in biology, such phase transitions and bifurcation phenomena are sometimes referred to as branching processes. We direct the reader to the Refs.^22,23^ for more on such branching processes in biology.

Suppose now that the ${\mathcal {R}}^{{\tilde{G}}}$-matrix associated with *T*(*t*), which we shall denote by ${\mathcal {R}}^{{\tilde{G}}}(t)$ has exactly one eigenvalue $\lambda (t)$ such that $\lambda (0)=1$ (this means that the algebraic multiplicity of $\lambda (t)$ is one). If we further assume that ${\dot{\lambda }}(0)\ne 0$, i.e $\lambda (t)$ crosses 1 transversely at $t=0$. Then, the computation (12) shows that at $t=0$ the map $d{\mathcal {G}}_0$ has a 1-dimensional kernel and cokernel and that $(\partial _t|_{t=0} d{\mathcal {G}}_0)(\ker (d{\mathcal {G}}_0)) \notin \mathop {\mathrm {im}}(d{\mathcal {G}}_0)$. Then, the Crandall–Rabinowitz theorem (Theorem 1.7. in^24^) shows that a phase transition must occur at $t=0$. We shall state this separately as follows.

### Proposition 3

*Consider a* 1-*parameter family of parameters* $T(t)=(T_1(t), \ldots , T_n(t))$ *whose associated* ${\mathcal {R}}^{{\tilde{G}}}$-*matrix has a unique eigenvalue* $\lambda (t)$ *satisfying* $\lambda (0)=1$. *If* ${\dot{\lambda }}(0) \ne 0$, *then a phase transition must occur at* $t=0$.

### Example 4

($n=2$) We have $\tilde{G_i}(x_1,x_2)=\frac{G_i'(x_i)}{G_i'(1)} G_j(x_j)$ for $i=1,2$ and $j\ne i$. In particular,

$$\begin{aligned} \frac{\partial \tilde{G_1}}{\partial x_1} = \frac{G_1''(x_1)}{G_1'(1)} G_2(x_2), \quad \frac{\partial \tilde{G_1}}{\partial x_2} = \frac{G_1'(x_1)G_2'(x_2)}{G_1'(1)}, \end{aligned}$$

and similarly for $\tilde{G_2}$. Hence, the ${\mathcal {R}}^1$-matrix can be written as

$$\begin{aligned} {\mathcal {R}}^{{\tilde{G}}} = \begin{pmatrix} T_1\frac{G_1''(1)}{G_1'(1)} &{}\quad T_1 G_1'(1) \\ T_2 G_2'(1) &{}\quad T_2\frac{G_2''(1)}{G_2'(1)} \end{pmatrix} = \begin{pmatrix} T_1 \frac{ {\mathbf{E}}(k_1^2)- {\mathbf{E}}(k_1)}{{\mathbf{E}}(k_1)} &{}\quad T_1 {\mathbf{E}}(k_1) \\ T_2 {\mathbf{E}}(k_2) &{}\quad T_2 \frac{ {\mathbf{E}}(k_2^2)- {\mathbf{E}}(k_2)}{{\mathbf{E}}(k_2)} \end{pmatrix}. \end{aligned}$$

### Example 5

($n=2$ *with 2 neighbors each*) Consider $T_1<T_2$ and $G_1(x)=x^2$, $G_2(x)=x^2$. Then, we have $\tilde{G_i}(x_1,x_2)=x_i x_j^2$ for $i=1,2$ and $j\ne i$. Then, the ${\mathcal {R}}^{{\tilde{G}}}$-matrix is

$$\begin{aligned} {\mathcal {R}}^{{\tilde{G}}} = \begin{pmatrix} T_1 &{}\quad 2T_1 \\ 2T_2 &{}\quad T_2 \end{pmatrix}, \end{aligned}$$

whose eigenvalues are

$$\begin{aligned} \lambda _{\pm } = \frac{T_1+T_2}{2} + \frac{1}{2} \sqrt{T_1^2 +T_2^2 + 14 T_1 T_2}. \end{aligned}$$

Clearly, $\lambda _-<0$ and so a phase transition must occurs when $\lambda _+$ crosses 1, i.e. when

$$\begin{aligned} T_1+T_2 + \sqrt{T_1^2 +T_2^2 + 14 T_1 T_2}=2. \end{aligned}$$

Furthermore, in this case the equations for $(q_1,q_2)$ read

$$\begin{aligned} q_1&= 1-T_1 + T_1 q_1 q_2^2 \\ q_2&= 1-T_2 + T_2 q_1^2 q_2 . \end{aligned}$$

In particular, using the first equation to write $q_1$ in terms of $q_2$ and inserting in the second we find that solutions are given by $q_2=1$ and solutions of the quartic equation

$$\begin{aligned} T_1^2 q_2^4+T_1^2T_2 q_2^3 + (T_2T_1-2) T_1 q_2^2 + T_1 T_2 (T_1-2) q_2 -T_2 +1 =0. \end{aligned}$$

### Example 6

($n=2$ *with 2 exponentially distributed graphs*) Again, we consider $T_1<T_2$ and $G_1(x)=x^{-N_1(1-x)}$, $G_2(x)=x^{-N_2(1-x)}$ for $N_1>N_2$. In this situation we have

$$\begin{aligned} \tilde{G_1}(x_1,x_2)=e^{-N_1(1-x_1)-N_2(1-x_2)}=\tilde{G_2}(x_1,x_2). \end{aligned}$$

Then, the ${\mathcal {R}}^1$-matrix is

$$\begin{aligned} {\mathcal {R}}^{{\tilde{G}}} = \begin{pmatrix} T_1N_1 &{}\quad T_1N_1 \\ T_2N_2 &{}\quad T_2N_2 \end{pmatrix}, \end{aligned}$$

whose eigenvalues are 0 and

$$\begin{aligned} \lambda = T_1N_1+T_2N_2. \end{aligned}$$

which in this case coincides with $R_0$ and we therefore find that a phase transition occurs when $R_0$ crosses 1. In fact, it is tempting to regard $T_1N_1$ and $T_2N_2$ as the respective contributions to $R_0$ by the networks ${\mathcal {G}}_1$ and ${\mathcal {G}}_2$. Indeed, $R_0(1)= 2T_1N_1$ and $R_0(2)= 2T_2N_2$ so that

$$\begin{aligned} R_0=\frac{1}{2} (R_0(1)+R_0(2)), \end{aligned}$$

as shown in Remark 8.

These interpretations of $R_0(1)$ and $R_0(2)$ may, however, not be appropriate to interpret some non-intuitive phenomena. For example, one may be lead to think that both $q_1$ and $q_2$ are non-increasing with respect to $R_0(1)$ and $R_0(2)$. However, this need not be true as we shall illustrate in an example. In this case the equations for $(q_1,q_2)$ read

$$\begin{aligned} q_1&= 1-T_1 + T_1 e^{-N_1(1-q_1)-N_2(1-q_2)} \\ q_2&= 1-T_2 + T_2 e^{-N_1(1-q_1)-N_2(1-q_2)} , \end{aligned}$$

and we shall now iterate the right hand side to approximate two solutions. Say, in the case when $N_1=50, T_1=0.1$ and $N_2=2, T_2=0.6$, which corresponds to $R_0(1)=5$ and $R_0(2)=1.2$ we have $(q_1,q_2)\sim (0.9,0.4)$. On the other hand, if instead $N_1=5,T_1=0.2$, which corresponds to $R_0(1)= 1$, while the rest remains as before, we have $(q_1,q_2) \sim (0.83 , 0.49)$ and so $q_1$ did decrease but $q_2$ increased.

However, this situation is not totally counter-intuitive as overall, the value of ${\mathbf{P}}$, the probability that a random vertex escapes infection, does increase in the second example where ${\mathbf{P}}\sim 0.17$ in comparison with a ${\mathbf{P}}\sim 0.13$ in the first example.

## Dynamic modeling in a local mean field approximation

In section “Prevalence of infection and percolation (the late stages of an outbreak)” we studied a mathematical framework, related to percolation models, and used the properties of the network in order to compute the probability that a given node will eventually be infected. However, this framework does not look at the specific way the infection propagates in the network through time. That will be the topic of the current section. Here, we investigate an extended SIR type system modeling the spread of an epidemic on a network with different types of contacts, meaning that the transmissibilities are not all the same and can be gauged to approximate different kinds of contacts. As before, we consider a set of *n* graphs ${\mathcal {G}}_1, \ldots , {\mathcal {G}}_n$ with the same vertices but different edges. These encode the interactions and each graph ${\mathcal {G}}_i$ is weighted by a transmissibility per unit time $\beta _i$ encoding the probability of transmitting the disease through that interaction (per time unit). Our model is a simple alternative to^25^ which more directly deals with contact duration.

### The model

For each vertex *v* of ${\mathcal {G}}$ we shall denote by ${\mathcal {G}}_i(v)$ all its neighbors through the graph ${\mathcal {G}}_i$, in other words ${\mathcal {G}}_i(v)$ is the set of all vertices which are connected to *v* through a an edge of ${\mathcal {G}}_i$. Then, we respectively denote by $s_v$, $x_v$ and $r_v$ the probabilities that *v* is either susceptible to the disease, infected, or removed. The dynamics of this network SIR model is then approximately governed by

13 $$\begin{aligned} \dot{s_v}&= - \sum _{i=1}^n \beta _i \sum _{w \in {\mathcal {G}}_i(v)} s_v x_w \nonumber \\ \dot{x_v}&= - \gamma x_v + \sum _{i=1}^n \beta _i \sum _{w \in {\mathcal {G}}_i(v)} s_v x_w \nonumber \\ \dot{r_v}&= \gamma x_v , \end{aligned}$$

where $\gamma >0$ is the rate of recovery.

#### Remark 11

Alternatively, we can let $A^{i}_{vw}$ be the entries of the adjunction matrix of the graph ${\mathcal {G}}_i$ and write the sum $\sum _{w \in {\mathcal {G}}(v_i)} s_v x_w$ as $\sum _{w} A^{i}_{vw}s_v x_w$.

#### Remark 12

The system (13) is an approximation because the average probability that *v* is susceptible and *w* infected is only approximately given by $s_v x_w$. In order to work with a non-approximate model one would have to write an infinite array of equations modeling the dynamics of the average probabilities of all such nonlinear quantities. See also^3^ for such an analysis carried out in the situation where there is only one type of interaction.

### Classifying the disease free equilibrium

It is clear from the equation $\dot{r_v}=\gamma x_v$ that any equilibrium solution of the system (13) we must have $x_v=0$ for all *v*. Hence, any equilibrium solution is disease free. In fact, setting $x_v=0$ and $s_v+r_v=1$ gives a 1-parameter family of equilibrium solutions of the system and any equilibrium solution must be one of these.

An important question is then to understand if a non-constant solution converges to one of these equilibria and to which? The fact that any solution $\lbrace (s_v(t),x_v(t),r_v(t)) | \ t \in [0,+\infty ) \rbrace _{v}$ converges to an equilibrium is immediate from the fact that $r_v(t)$ is nondecreasing and bounded, hence the limit

$$\begin{aligned} r_v(\infty ):=\lim _{t \rightarrow +\infty } r_v(t) \in [0,1] \end{aligned}$$

exists. Furthermore $\lim _{t \rightarrow + \infty } \dot{r_v}=0$ which implies $x_v(\infty )=0$ as we wanted to show. The main question then becomes:

**Question 1 **What is the disease free equilibrium $(s_v(\infty ),0,r_v(\infty ))$ to which a solution starting at $(s_v(0),x_v(0),r_v(0))$ converges? Can we understand how this depends on the properties of ${\mathcal {G}}_1, \ldots , {\mathcal {G}}_n$ and $\beta _1, \ldots , \beta _n$?

From the system of equations (13) we find that for each vertex *v* of ${\mathcal {G}}$, there are two conserved quantities

$$\begin{aligned} 1=s_v+x_v+r_v, \end{aligned}$$

and

$$\begin{aligned} H(v):= s_v \exp \left( \frac{1}{\gamma } \sum _{i=1}^n \beta _i \sum _{w \in {\mathcal {G}}_i(v)} r_w \right) . \end{aligned}$$

Let *N* be the total number of vertices. These conserved quantities reduces the system of 3*N* equations for 3*N* functions to a system involving only *N* functions. In fact introducing $s_v=1-r_v-x_v$ and the last equation of system (13) into the previous conserved quantity we find that

$$\begin{aligned} H(v)= \left( 1- r_v - \frac{\dot{r_v}}{\gamma } \right) \exp \left( \frac{1}{\gamma } \sum _{i=1}^n \beta _i \sum _{w \in {\mathcal {G}}_i(v)} r_w \right) . \end{aligned}$$

Suppose *v* starts susceptible, then $\dot{r_v}(0)=0=r_v(0)$ and so $H(v)=1$, from which we have

$$\begin{aligned} \left( 1- r_v - \frac{\dot{r_v}}{\gamma } \right) \exp \left( \frac{1}{\gamma } \sum _{i=1}^n \beta _i \sum _{w \in {\mathcal {G}}_i(v)} r_w \right) =1 \end{aligned}$$

for all time. Given that the solution converges to an equilibrium, we must have $\lim _{t \rightarrow + \infty } \dot{r_v}=0$ and so

14 $$\begin{aligned} 1=\left( 1- r_v \right) \exp \left( \frac{1}{\gamma } \sum _{i=1}^n \beta _i \sum _{w \in {\mathcal {G}}_i(v)} r_w \right) , \end{aligned}$$

where in this equation we have written $r_v(\infty )$ as $r_v$ to simplify notation. It will prove convenient to have the following notion at hand.

#### Definition 3

Let *v* be a vertex of ${\mathcal {G}}$ and $k_i(v)$ its degree as a vertex of ${\mathcal {G}}_i$ we shall denote by

$$\begin{aligned} R(v):=\sum _{i=1}^n \frac{\beta _i }{\gamma } k_i(v) \end{aligned}$$

the expected number of infections *v* will cause if infected.

#### Remark 13

Notice that the quantity $\beta _i/\gamma$ represents the probability that an interaction of ${\mathcal {G}}_i$, between an infected and a susceptible individual, results in an infection. Hence, the quantity $\sum _{i=1}^n \frac{\beta _i k_i(v)}{\gamma }$ can be regarded as the average number of infections the individual represented by *v* is expected to cause if it is infected.

#### Remark 14

For example, let us assume we have a sufficiently simple situation so that $r_w \approx r_v$ for all $w \in {\mathcal {G}}_i(v)$. Then, inserting this into (14) we find that

$$\begin{aligned} 1=\left( 1- r_v \right) \exp \left( r_v \sum _{i=1}^n \frac{\beta _i}{\gamma } k_i(v) \right) = \left( 1- r_v \right) \exp \left( r_v R(v) \right) . \end{aligned}$$

In this situation, the right hand side equals 1 when $r_v=0$ and vanishes when $r_v=1$. Hence, by the mean value theorem, a solution with $r_v \in (0,1)$ exists if and only if the derivative of the right hand at $r_v=0$, is positive. Such a derivative can be computed to be $R(v) -1$ which is positive if and only if $R(v)>1$.

In order to investigate the existence of solutions to Eq. (14) with $r_v \ne 0$ it is convenient to rewrite this equation as

15 $$\begin{aligned} r_v= 1-\exp \left( - \sum _{i=1}^n \frac{\beta _i}{\gamma } \sum _{w \in {\mathcal {G}}_i(v)} r_w \right) = 1-\exp \left( - \sum _{i=1}^n \frac{\beta _i}{\gamma } \sum _{w} A^i_{vw} r_w \right) . \end{aligned}$$

Hence, our problem is now recast as the problem of looking for fixed points of the function $F:[0,1]^N \rightarrow [0,1]^N$ given by $F(r_1, \ldots , r_N)=(F_1(r_1, \ldots , r_N) , \ldots , F_N(r_1, \ldots , r_N))$ where

$$\begin{aligned} F_v(r_1, \ldots , r_N)=1-\exp \left( - \sum _{i=1}^n \frac{\beta _i}{\gamma } \sum _{w} A^i_{vw} r_w \right) . \end{aligned}$$

The first obvious fixed point is that occurring at the origin which corresponds to all individuals still being susceptible, i.e. the disease never spread. In the next result we give a criteria for the existence of another fixed point.

#### Theorem 1

*Suppose that for all vertices* *v* *of* ${\mathcal {G}}$ *we have* $R(v) >1$. *Then, the solution to the system* (13) *starting with* $r_v(0) =0$ *converges to a disease free equilibrium with* $r_v \ne 0$ *for all* *v*. *This satisfies Eq.* (15) *and the bound*

$$\begin{aligned} r_v \le 1 - \exp \left( -R(v)\right) . \end{aligned}$$

*In particular, we find that the upper bound is increasing with* *R*(*v*) (*in agreement with basic intuition*).

#### Proof

The proof that the solution converges to a disease free equilibrium is given in the beginning of this subsection. This must satisfy (15) and we shall now prove that, under the conditions stated, $r_v \in (0,1)$, i.e. $r_v \ne 0$. We proceed as in the proof of Proposition 1 by applying Bower’s fixed point theorem. Let $\varepsilon >0$ to be fixed later, then the Taylor expansion of $F_v$ around the origin reads

$$\begin{aligned} F_v(\varepsilon , \ldots , \varepsilon ) = \sum _{p} \frac{\partial F_v}{\partial r_p} \varepsilon + O(\varepsilon ^2) = \left( \sum _{i=1}^n \frac{\beta _i}{\gamma } \sum _{p} A^i_{vp} \right) \varepsilon + O(\varepsilon ^2) = \sum _{i=1}^n \frac{\beta _i k_i(v)}{\gamma } \varepsilon + O(\varepsilon ^2) = R(v) \varepsilon + O(\varepsilon ^2). \end{aligned}$$

Furthermore, $F_v$ is non-decreasing with respect to each entry. Hence, if $R(v) >1$ for all *v* we find that for sufficiently small $\varepsilon >0$ the function $F|_{[\varepsilon ,1]^N}$ maps $[\varepsilon ,1]^N$ to itself and by the Brower fixed point theorem must have a fixed point in $[\varepsilon ,1]^N$.

We turn now to the proof of the upper bound for $r_v$ given in the statement. Recall that $k_i(v)$ denotes the degree of *v* in ${\mathcal {G}}_i$. From Eq. (14) we immediately find that the argument of the exponential in the right hand side satisfies

$$\begin{aligned} \frac{1}{\gamma } \sum _{i=1}^n \beta _i \sum _{w \in {\mathcal {G}}_i(v)} r_w \le \sum _{i=1}^n \frac{\beta _i}{\gamma } \sum _{w \in {\mathcal {G}}(v_i)} 1 = \sum _{i=1}^n \frac{\beta _i}{\gamma } k_i(v) . \end{aligned}$$

Hence, we discover that $r_v=r_v(\infty )$ satisfies

16 $$\begin{aligned} r_v \le 1 - \exp \left( -\sum _{i=1}^n \frac{\beta _i}{\gamma } k_i(v) \right) \end{aligned}$$

Inserting the Definition 3, of *R*(*v*), in this bound gives $r_v \le 1 - \exp \left( -R(v)\right)$, as claimed in the statement. $\square$

#### Remark 15

Inserting the equation $\dot{r_w}=\gamma x_w$ for $w \in {\mathcal {G}}_i(v)$, in the first equation of (13) we find that

$$\begin{aligned} \dot{s_v} = - \left( \sum _{i=1}^n \frac{\beta _i}{\gamma } \sum _{w \in {\mathcal {G}}_i(v)} \dot{r_w} \right) s_v. \end{aligned}$$

this can be integrated using the initial condition $r_w(0)=0$ to obtain

$$\begin{aligned} s_v(t) = s_v(0) \exp \left( - \sum _{i=1}^n \frac{\beta _i}{\gamma } \sum _{w \in {\mathcal {G}}_i(v)} r_w(t) \right) . \end{aligned}$$

This is a very nice and beautiful formula, but it is not of much use if we know nothing about $r_w(t)$. However, we do can take the limit as $t \rightarrow + \infty$ and assume we are converging to a disease free equilibrium $(s_v,x_v,r_v)=(s_v,0,r_v)$ whose existence is assured, for example, under the assumptions of Proposition 1. In such a situation we have $\lim _{t \rightarrow + \infty } s_v(t) =1- \lim _{t \rightarrow + \infty } r_v(t)$. Hence, in this limit the previous equation turns into

$$\begin{aligned} 1-r_v(\infty ) = s_v(0) \exp \left( - \sum _{i=1}^n \frac{\beta _i}{\gamma } \sum _{w \in {\mathcal {G}}_i(v)} r_w \right) . \end{aligned}$$

By setting $s_v(0)=1$ this is the same equation which we have previously derived. This situation is slightly more general than the one we have analyzed in Theorem 1 and a similar results hold yielding the bound

$$\begin{aligned} r_v(\infty ) \le 1 - s_v(0) \exp \left( - R(v) \right) . \end{aligned}$$

## Dynamic modeling in a mean field approximation by degree similarity

The system of the previous section, though very general is extremely large and difficult to investigate. For this reason, it is convenient to find simpler systems which we can more easily analyze. This is the content of this section where we will consider a system for the average probability that vertices of a given degree are in specific states. This is an oversimplified assumption which nevertheless allows one to gain a lot of insight on the dynamics of an epidemic in a network. The approach we take here is mostly inspired from that of^2^, where the authors first learned it for the case $n=1$. There are also interesting individual based approaches which however require knowing the whole network structure and they also only have $n=1$, see for example^26^. See also^27^ for some computational results using a model on a weighted network.

### The model

For each $k=(k_1 , \ldots , k_n)$ we shall denote by $s_k$, $x_k$ and $r_k$ the average probabilities that a vertex with degree *k* is susceptible, infected and removed respectively. Such a network SIR model is governed by the following system

17 $$\begin{aligned} \dot{s_k}&= - \sum _{i=1}^n \beta _i k_i v_i s_k \nonumber \\ \dot{x_k}&= - \gamma x_k + \sum _{i=1}^n \beta _i k_i v_i s_k \nonumber \\ \dot{r_k}&= \gamma x_k , \end{aligned}$$

where for $$i=1 , \ldots , n$$

$$\begin{aligned} v_i := \sum _{k_1 , \ldots , k_n} {\tilde{P}}_i(k_1 , \ldots , k_n) x_{(k_1 , \ldots , k_i +1 , \ldots , k_n)}, \end{aligned}$$

is the averaged probability of an individual being infected after following a random edge of the graph ${\mathcal {G}}_i$.

#### Remark 16

Again, in writing the system (17) we have used one large simplification. Namely, we have approximated the average value of $x_{k'}s_k$ by the product of the average values of $x_{k'}$ and $s_k$.

### An equivalent (much reduced) system of equations

Notice that (17) can potentially be an extremely large system, as there are as many groups of 3-equations as degree combinations. It is remarkable that this system can be extremely reduced to one that only involves *n* equations for *n* functions. For this, it is convenient to introduce quantities $w_i$ measuring the average number of removed individuals following an edge of ${\mathcal {G}}_i$. These are given by

$$\begin{aligned} w_i := \sum _{k_1 , \ldots , k_n} {\tilde{P}}_i(k_1 , \ldots , k_n) r_{(k_1 , \ldots , k_i +1 , \ldots , k_n)} , \end{aligned}$$

for $i=1 , \ldots , n$. Then, we have $\dot{w_i}=\gamma v_i$ which inserting into the equation for $\dot{s_k}$ yields $\dot{s_k}= - \frac{1}{\gamma } \sum _{i=1}^n \beta _i k_i \dot{w_i} s_k$. Assuming that the epidemic outbreak starts with no-one removed from previous infections, we have $w_i(0)=0$ (which follows from $r_k(0)=0$ for all $k \in {\mathbb {N}}^n$) and so

$$\begin{aligned} s_k(t) = s_k(0) \exp \left( - \sum _{i=1}^n \frac{\beta _i}{\gamma } k_i w_i \right) , \end{aligned}$$

which we can also rewrite as

$$\begin{aligned} s_k(t)= s_k(0) u_1^{k_1} \ldots u_n^{k_n}, \end{aligned}$$

for $u_i (t) = \exp \left( - \frac{\beta _i}{\gamma } w_i \right)$. Now, recall that $s_{k}+x_{k}+r_{k}=1$ and so

18 $$\begin{aligned} v_i&= \sum _{k_1 , \ldots , k_n} {\tilde{P}}_i(k_1 , \ldots , k_n) (1 - r_{(k_1 , \ldots , k_i +1 , \ldots , k_n)} - s_{(k_1 , \ldots , k_i +1 , \ldots , k_n)} ) \nonumber \\&= 1- \sum _{k_1 , \ldots , k_n} {\tilde{P}}_i(k_1 , \ldots , k_n) r_{(k_1 , \ldots , k_i +1 , \ldots , k_n)} \nonumber \\&\quad - \sum _{k_1 , \dots , k_n} {\tilde{P}}_i(k_1 , \ldots , k_n) s_{(k_1 , \ldots , k_i+1, \ldots , k_n)}(0) u_1^{k_1} \ldots u_{i}^{k_i+1} \ldots u_n^{k_n} \nonumber \\&= 1 - w_i - {\tilde{H}}_i(u_1, \ldots , u_n), \end{aligned}$$

where in the last equality we have used the generating function (6) whose definition we recall to be

$$\begin{aligned} {\tilde{H}}_i(x_1, \ldots , x_n)= \sum _{k_1 , \dots , k_n} {\tilde{P}}_i(k_1 , \ldots , k_n) s_{(k_1 , \ldots , k_i+1 , \ldots , k_n)}(0) x_1^{k_1} \ldots x_i^{k_i+1} \ldots x_n^{k_n}, \end{aligned}$$

which depends on the initial conditions $s_{(k_1 , \ldots , k_n)}(0)$.

#### Remark 17

Consider the case where one is willing to make the simplifying assumption that $s_k(0)=s(0)$ for all $k \in {\mathbb {N}}^n$, i.e. the initial proportion of susceptible individuals is independent of their degree distribution (this may be a reasonable assumption for a new disease which begins to spread in an unknown part of the population). Then, ${\tilde{H}}_i(x_1, \ldots , x_n)=s(0) {\tilde{G}}_i(x_1, \ldots , x_n)$ for all $i=1, \ldots , n$. Notice a few properties of the function ${\tilde{H}}$, namely it is nondecreasing with respect to each coordinate, and

$$\begin{aligned} {\tilde{H}}_i(1)= \sum _{k_1 , \dots , k_n} {\tilde{P}}_i(k_1 , \ldots , k_n) s_{(k_1 , \ldots , k_i+1 , \ldots , k_n)}(0)= {\mathbf{E}}[s(0) | {\mathcal {G}}_i], \end{aligned}$$

i.e. the average number of initially susceptible individuals in ${\mathcal {G}}_i$, meaning those which have edges in ${\mathcal {G}}_i$.

Using $v_i= \frac{1}{\gamma }\dot{w_i}$ and $w_i = - \frac{\gamma }{\beta _i} \log u_i$ and rearranging, we find that (17) can be written as a system for $w=(w_1, \ldots , w_n)$ given by

19 $$\begin{aligned} \gamma ^{-1} \dot{w_i}&= 1 - w_i - {\tilde{H}}_i \left( e^{-\frac{\beta _1}{\gamma } w_1}, \ldots , e^{-\frac{\beta _n}{\gamma } w_n}\right) . \end{aligned}$$

and further using and $w_i = - \frac{\gamma }{\beta _i} \log u_i$ this can be rewritten as a system for $u=(u_1, \ldots , u_n)$

20 $$\begin{aligned} \dot{u_i}&= - \beta _i u_i \left( 1 + \frac{\gamma }{\beta _i} \log u_i - {\tilde{H}}_i(u_1, \ldots , u_n) \right) , \end{aligned}$$

which is a substantially smaller than the initial one in (17).

### Existence of an equilibrium

At an equilibrium point we either have the rights hand side of (19) vanishing, i.e.

$$\begin{aligned} 1 - w_i - {\tilde{H}}_i \left( e^{-\frac{\beta _1}{\gamma } w_1}, \ldots , e^{-\frac{\beta _n}{\gamma } w_n}\right) =0 , \end{aligned}$$

or, written as a fixed point equation, as

21 $$\begin{aligned} w_i=1 - {\tilde{H}}_i \left( e^{-\frac{\beta _1 w_1}{\gamma }}, \ldots , e^{-\frac{\beta _n w_n}{\gamma }} \right) , \end{aligned}$$

for $i=1, \ldots , n$. In particular, notice from Eq. (18) that this implies that $v_i=0$ for all $i=1, \ldots , n$ and so this equation encodes a disease free equilibrium to which the solution of the system is expected to converge.

As in section “Prevalence of infection and percolation (the late stages of an outbreak)”, we shall start by analyzing the case when $n=1$ in the following example. It will serve as a good exercise for the $n>1$ case. See also^2^ for this analysis in the case $n=1$.

#### Example 7

($n=1$) In this situation, there is only one ${\tilde{H}}_i(x)$ which is given by

$$\begin{aligned} {\tilde{H}}(x) = \sum _{k=0}^{+\infty } {\tilde{P}}(k) s_{k+1}(0) x^{k+1}= \frac{1}{{\mathbf{E}}(k)} \sum _{k=1}^{+\infty } k P(k) s_{k}(0) x^{k}. \end{aligned}$$

and the Eq. (21) for an equilibrium is

22 $$\begin{aligned} w=1-{\tilde{H}}(e^{-\frac{\beta w}{\gamma }} ). \end{aligned}$$

Hence, an equilibrium is determined by fixed points of the function *g*(*w*) given by the right hand side of (22). Notice that $g(0)=1-{\mathbf{E}}[s(0)]>0$ (even though it may be very small) while $g(1)=1-{\tilde{H}}(e^{-\frac{\beta }{\gamma }} ) <1$, and so a fixed point $w^{eq}$ always exist by the intermediate value theorem. Moreover, we find from

$$\begin{aligned} g'(w)&= \frac{\beta }{\gamma } {\tilde{H}}'(e^{-\frac{\beta w}{\gamma }}) >0, \end{aligned}$$

that *g*(*w*) is increasing while from

$$\begin{aligned} g''(w)&= - \left( \frac{\beta }{\gamma } \right) ^2 {\tilde{H}}''(e^{-\frac{\beta w}{\gamma }}) <0, \end{aligned}$$

we find that its concavity always faces down. Hence, the equilibrium point $w^{eq}$ must be unique. We further deduce the equilibrium value of $s_k$ to be

$$\begin{aligned} s_k^{eq} = s(0) e^{-\frac{k\beta }{\gamma } w^{eq} }. \end{aligned}$$

However, when ${\mathbf{E}}[s(0)]=1$, then there is fixed point of *g* at $w=0$ which corresponds to never having a disease spreading. For another fixed point $w^{eq} \in (0,1)$ to exist we must have $g'(0)>1$ which can be written as

$$\begin{aligned} \frac{\beta }{\gamma } {\tilde{H}}'(1)>1, \end{aligned}$$

and can be identified with the initial basic reproduction number. This situation with ${\mathbf{E}}[s(0)]=1$ is relevant, for example, when considering an approximately infinite number of individuals with only a finite number of infected individuals.

#### Example 8

($n=1$ *and every vertex with k neighbors*) In this case ${\tilde{H}}(x)=x^k$ and so the equilibrium is attained at a $w^{eq}=w$ such that $w=1-s(0) e^{ -\frac{\beta }{\gamma } k w}$.

Also in this case, the insights given by the previous example can be extended to higher dimensions to prove the existence of an equilibrium point of the system starting at a given configuration of initially susceptible individuals.

#### Proposition 4

*Suppose that the average number of initially susceptible individuals* ${\mathbb {E}}[s(0)]<1$. *Then, there is an equilibrium point* $w^{eq}=(w_1^{eq}, \ldots , w_n^{eq}) \in (0,1)^n$ *such that*

$$\begin{aligned} s_k^{eq}=s_k(0) \exp \left( - \sum _{i=1}^n \frac{\beta _i}{\gamma } k_i w_i^{eq} \right) . \end{aligned}$$

*In particular*,

$$\begin{aligned} r_k^{eq}=1-s_k(0) \exp \left( - \sum _{i=1}^n \frac{\beta _i}{\gamma } k_i w_i^{eq} \right) \le 1-s_k(0) \exp \left( - \sum _{i=1}^n \frac{\beta _i}{\gamma } k_i \right) \end{aligned}$$

#### Proof

We are looking for fixed points of the (continuous) map $g: [0,1]^n \rightarrow [0,1]^n$ is given by

$$\begin{aligned} g(w_1 , \ldots , w_n) = \left( 1 - {\tilde{H}}_1 \left( e^{-\frac{\beta _1 w_1}{\gamma }}, \ldots , e^{-\frac{\beta _n w_n}{\gamma }} \right) , \ldots , 1 - {\tilde{H}}_n \left( e^{-\frac{\beta _1 w_1}{\gamma }}, \ldots , e^{-\frac{\beta _n w_n}{\gamma }} \right) \right) , \end{aligned}$$

and again at least one such equilibrium $w^{eq}=(w_1^{eq} , \ldots , w_n^{eq})$ exists from Brower’s fixed point theorem. Using this we find

$$\begin{aligned} s_k^{eq}=s_k(0) \exp \left( - \sum _{i=1}^n \frac{\beta _i}{\gamma } k_i w_i^{eq} \right) , \end{aligned}$$

which again we can see to be exponentially decreasing with $k_i$ and $T_i$ (notice however that the $w_i^{eq}$ themselves are also functions of the $\beta _i/\gamma$ so this statement is somewhat imprecise).

Given that at the equilibrium point all $x_k=0$ (recall that $\gamma v_i=\dot{w_i}$) we have $s_k^{eq}+r_k^{eq}=1$ from which the last equality and inequality in the statement follow. $\square$

#### Remark 18

Of course, when ${\mathbb {E}}[s(0)]=1$ there is an equilibrium point with $w^{eq}=0$ and $s_k^{eq}=1$.

### Convergence to the equilibrium

We consider the cases $n=1$ and $n=2$ for which we have the flow equation (20) which we rewrite as $\dot{u_i}=f_i(u_1 , \ldots , u_n)$, for

23 $$\begin{aligned} f_i(u_1 , \ldots , u_n)&= - \beta _i u_i \left( 1 + \frac{\gamma }{\beta _i} \log u_i - s(0) {\tilde{G}}_i(u_1, \ldots , u_n) \right) . \end{aligned}$$

In the case $n=1$ we have

$$\begin{aligned} f'(u)&= - \beta \left( 1 + \frac{\gamma }{\beta } \log u - s(0) {\tilde{G}}(u) \right) - \beta u \left( \frac{\gamma }{\beta } \frac{1}{u} - s(0) {\tilde{G}}'(u) \right) \\&= -\beta - \gamma \log u + s(0) T {\tilde{G}}(u) - \gamma + s(0) u \beta {\tilde{G}}'(u) . \end{aligned}$$

In particular, $\lim _{u \rightarrow 0^+} f(u)=0$ with $\lim _{u \rightarrow 0^+} f'(u) = + \infty$ and so there is $\varepsilon >0$ such that $f(u)>0$ in $(0,\varepsilon )$ . On the other hand while $\lim _{u \rightarrow 1^-}f(u)=- \beta \left( 1 - s(0)\right) <0$. Hence, the solution to (20) with $n=1$ stays bounded inside (0, 1) and therefore converges to an equilibrium point $u^{eq} \in (0,1)$ with $f(u^{eq})=0$. Hence,

$$\begin{aligned} f'(u^{eq})&= - \beta u \left( \frac{\gamma }{\beta } \frac{1}{u} - s(0) {\tilde{G}}'(u) \right) = - \left( \gamma - \beta s(0) {\tilde{G}}'(u) u \right) . \end{aligned}$$

We now turn to the case when $n=2$. In this situation we have $\lim _{u_i \rightarrow 0^+} f_i(u_1,u_2) =0$ and

$$\begin{aligned} \frac{\partial f_1}{\partial u_1}&= - \beta _1 \left( 1 + \frac{\gamma }{\beta _1} \log u_1 - s(0) {\tilde{G}}_1(u_1 , u_2) \right) - \beta _1 u_1 \left( \frac{\gamma }{\beta _1} \frac{1}{u_1} - s(0) \frac{\partial {\tilde{G}}_1}{\partial x_1}(u_1, u_2) \right) \\&= - \beta _1 - \gamma \log u_1 - s(0) \beta _1{\tilde{G}}_1(u_1 , u_2) - \gamma - s(0) u_1 \beta _1 \frac{\partial {\tilde{G}}_1}{\partial x_1}(u_1, u_2) , \end{aligned}$$

and similarly for $\frac{\partial f_2}{\partial u_2}$. In particular, we find that $\lim _{u_i \rightarrow 0^+} f_i(u_1,u_2) = + \infty$. Hence, we have that there is $\varepsilon >0$ such that $f_i(u_1,u_2)>0$ for $u_i \in (0,\varepsilon )$. Furthermore,

$$\begin{aligned} \lim _{u_1 \rightarrow 1^-} f_1 (u_1,u_2)&= - \beta _1 \left( 1 - s(0) {\tilde{G}}_1(1 , u_2) \right) \\&= - \beta _1 \left( 1 - s(0) G_2(u_2) \right) \\&\le - \beta _1 \left( 1 - s(0) \right) <0. \end{aligned}$$

Hence, the solutions stay inside the square $[0,1]^2$ and by the Poincaré–Bendixon theorem, it must therefore converge to an equilibrium point $(u_1^{eq},u_2^{eq})$ with $f_i(u_1^{eq},u_2^{eq})=0$ for $i=1,2$. Notice that here we have implicitly used the fact that there are no non-constant periodic solutions. That can easily be proven by looking at the system (17) from which we find that the $r_k$ must be non-decreasing and can only be constant at a disease free equilibrium.

### Short time behavior

Recall from Eq. (19) that the system is governed by the set of ordinary differential equations

24 $$\begin{aligned} \gamma ^{-1} \dot{w_i}&= 1 - w_i - {\tilde{H}}_i \left( e^{-\frac{\beta _1}{\gamma } w_1}, \ldots , e^{-\frac{\beta _n}{\gamma } w_n}\right) , \end{aligned}$$

for $i=1, \ldots , n$. Given that initially we have $w_i(0)=0$ we may Taylor expand right hand side above using ${\tilde{H}}_i(1)={\mathbf{E}}[s(0)]$ as alluded to in remark 17. This gives

$$\begin{aligned} \gamma ^{-1}\dot{w_i}&= 1 - w_i - \left( {\tilde{H}}_i(1) - \sum _{j=1}^n \frac{\partial {\tilde{H}}_i}{\partial x_j}\Big \vert _{x=1} \frac{\beta _j}{\gamma }w_j \right) + \cdots \\&= 1- {\mathbf{E}}[s(0)] + \sum _{j=1}^n \left( \frac{\beta _j}{\gamma } \frac{\partial {\tilde{H}}_i}{\partial x_j} \Big \vert _{x=1} - \delta _{ij} \right) w_j + \cdots \\&= 1- {\mathbf{E}}[s(0)] + \sum _{j=1}^n \left( {\mathcal {R}}^{{\tilde{H}}}_{ij} - \delta _{ij} \right) w_j + \cdots \end{aligned}$$

where ${\mathcal {R}}_{ij}^{H}$ is the (*i*, *j*) entry of the ${\mathcal {R}}^{{\tilde{H}}}$-matrix associated with the transmissibilities $T_k=\beta _k/\gamma$ as in definition 2, and the $\ldots$ denote terms of order $O(|w|^2)$. In this way, the above equation may be written as

25 $$\begin{aligned} \gamma ^{-1} {\dot{w}} = 1- {\mathbf{E}}[s(0)] + ({\mathcal {R}}^{{\tilde{H}}}-{\mathbb{I}}_{n,n}) \ w + \cdots \end{aligned}$$

Suppose that ${\mathcal {R}}^{{\tilde{H}}}$ can be diagonalised and let with eigenbasis $v_1, \ldots , v_n$ and $\lambda _1 \le \cdots \le \lambda _n$ the corresponding eigenvalues. Then, we write $w(t)= \sum _{l=1}^n w^l(t) v_l$ we find that each $w^l$ must solve

$$\begin{aligned} \dot{w^l}&= 1- {\mathbf{E}}[s(0)] + (\lambda _l - 1) w^l + \cdots , \end{aligned}$$

which we can integrate to obtain

$$\begin{aligned} w(t) = (1- {\mathbf{E}}[s(0)]) \sum _{l=1}^n \frac{1}{\lambda _l -1} \left( e^{(\lambda _l - 1)t} - 1 \right) . \end{aligned}$$

We therefore find that the initial exponential growth observed at the beginning of an outbreak is codified in the existence of an eigenvector of ${\mathcal {R}}^{{\tilde{H}}}$ greater than 1, as alluded to in the introduction.

## Major limitations

As with any model, those considered in this article have a scope and are therefore heavily limited. Obviously, there are limitations associated with the several assumptions and approximations made, but there are also several others. For instance, the fact that one does not need to consider the full form of the network, which can be interpreted as a strength of the model, may also be a severe limitation. Indeed, there are many different multi-graphs ${\mathcal {G}}={\mathcal {G}}_1 \cup \cdots \cup {\mathcal {G}}_n$ having the same degree distributions and the spread of an epidemic outbreak may have different features in such in-equivalent networks. This is not incorporated by our models which simply use the information on the degree distributions. However, as previously mentioned, this is limitation can also be seen positively. Indeed, while the exact form of the network is practically impossible to obtain in practice, estimating the degree distributions is a feasible endeavor. Nevertheless, one must bear such limitations into consideration anytime these models are used.

## Data Availability

All data generated or analysed during this study are included in this published article.
